# Severity and neurosurgical management of patients with traumatic spinal fractures in Saudi Arabia: a cross sectional study

**DOI:** 10.11604/pamj.2019.34.26.19354

**Published:** 2019-09-12

**Authors:** Khalid Hadi Aldosari, Yazeed Mohammed Aldhfyan, Mohammed H Karrar, Abdullah Mushabab Aldossary, Abdullah Abdulrahman Al Deailj, Khairat Hassan AL-Ameer, Munahi Lahiq Alsubaie

**Affiliations:** 1 Prince Sattam Bin Abdulaziz University, Colleges Of Medicine, Al-Kharj, Saudi Arabia; 2 Department Of Basic Medical Science, Colleges Of Medicine, Prince Sattam Bin Abdulaziz University, Al-Kharj 11942, Saudi Arabia; 3 Almaarefa University, College Of Medicine And Surgery, Riyadh, Saudi Arabia; 4 King Khalid University, College Of Medicine, Abha, Saudi Arabia

**Keywords:** Trauma, spinal fractures, neurosurgical management

## Abstract

**Introduction:**

Road traffic accidents (RTAs) are the most frequent cause of traumatic spinal injuries (TSIs), which account for up to 33.6% of all spinal fractures. The Kingdom of Saudi Arabia (KSA) is one of the countries which has high rates of SCIs and bears the economic burden of that situation.

**Methods:**

120 patients were included in this study, using a stringent set of inclusion and exclusion criteria. The patients were followed-up from the point of triage to admission and discharge. We analysed the clinical notes of the patients to determine the severity of their traumatic spinal injuries, the neurosurgical management carried out, and other prognosticating factors such as blood transfusion and the Glasgow Coma Scale (GCS). The data collected was analysed anonymously, and the confidentiality of all participants was respected.

**Results:**

Most of the patients were young adults and adolescents under the age of 40 (n = 96). There was a male preponderance of 84.1%. With respect to spinal injury stratification, 55 patients had cervical spine fractures, 10 patients had cervical lacerations, 85 patients had thoracolumbar spinal fractures, and 10 patients had thoracolumbar spinal lacerations. 35 patients had other fractures documented. All 120 patients were followed up to assess the management of their traumatic spinal injuries. 66.6% (n= 80) of all patients were managed conservatively, whereas the remaining 33.3% (n=40) were managed surgically.

**Conclusion:**

Trauma is an important cause of spinal injuries (TSIs), and untreated TSIs may lead to poor clinical outcome, especially if the cervical region is involved.

## Introduction

Traumatic spinal injuries (TSIs) are a frequent cause of morbidity and mortality among young adults, contributing to substantial individual impairment, as well as considerable psychological and socioeconomic burden globally [1]. It has been estimated that 768,473 cases of TSIs occur annually around the world [2]. Every year, approximately 25,000-50,000 people experience spinal cord injuries (SCIs) worldwide, 90% of which are caused by trauma [3]. According to a recent estimate, SCIs resulted in 1,000,000 years of healthy life loss among the American population in a 44-year time span [4]. Road traffic accidents (RTAs) are the most frequent cause of TSIs, which account for up to 33.6% of all spinal fractures [5]. The Kingdom of Saudi Arabia (KSA) is one of the countries which has high rates of SCIs and bears the economic burden of that situation. Management of TSIs or SCIs is a clinical challenge for neurosurgeons, as these injuries result in profound and long-term devastating physical and socio-economic consequences. The pathophysiology of SCIs is unique and critical, as the primary traumatic injury leads to progressive secondary insult in the form of ischemia, apoptosis and inflammation of the affected tissues [6]. Subsequently, the inflammatory mediators and cytotoxic debris add to the post-traumatic microenvironment, resulting in further loss of function and related complications. Therefore, the management of TSIs or SCIs is based on the management of the severity of the trauma and post-injury cascade of these events [6]. Standard management of TSIs or SCIs involves the protocols of basic life support (BSL), starting with the assessment of airway, breathing and circulation. Initial survey is followed by instant recognition of the spinal cord injury and rapid referral to the respective healthcare centers. Dealing with the patient suffering from a spinal injury is critical, as the treatment of such patients commences before the patient reaches the hospital. In this context, before handling or referring the patient to relevant centers, the immobilization of the injured part of the spine with the help of rigid cervical collar and backboard is very important because mishandling of patients with spinal cord trauma may worsen the injury, leading to poor outcome. It has been reported that 3-35% of spinal cord injuries develop after the primary injury during transportation and the early management [7]. Therefore, immobilization of the spine is the priority of pre-hospital management of the patients with spinal trauma. Studies have reported that spinal immobilization results in improved outcome. However, this spinal stabilization should not delay the treatment of the patient with life-threatening penetrating trauma, as immobilization delays the life-saving resuscitation.

On reaching the hospital, thorough neurological assessment is performed using different scales of grading for SCIs. After that, radiological assessment using radiological tools to evaluate the possible pathology of the trauma using imaging techniques is carried out. It has been reported that the patients with spinal cord injuries are at high risk of hypotension and hypoxemia due to hypovolemia secondary to polytrauma and decreased sympathetic tone [6]. Local hypoperfusion may result in spinal shock, leading to the loss of vascular tone and further low blood pressure and hypoperfusion [7,8]. Therefore, large volumes of crystalloids or Norepinephrine may be needed to maintain blood pressure. However, aggressiveness of the management depends upon the severity of the injury. Use of steroids is still controversial in patients suffering from SCIs. In addition to cardiovascular complications, thermoregulatory and bronchopulmonary derangements are also common in patients with SCIs [8]. Hence, such patients may develop life-threatening events and all the patients with spinal trauma are managed in the intensive care unit (ICU). Severe TSIs or SCIs may require surgical procedures. Recent advances have made it possible to fix and fuse nearly all TSIs effectively. Successful spinal trauma surgery advocates decompression of the injured neural bundles, reduction and fusion of spinal fractures, resulting in long-term stability. The timing of spinal surgery is controversial, as optimal timing of surgery has yet to be established. However, many surgeons prefer early decompression to reduce spinal cord compression, as recent studies have reported improved neurological outcome and reduced post-surgical complications with early surgeries [9,10]. Severity of TSIs can be assessed by the measurement of injury severity score (ISS), Glasgow Coma Scale (GCS) and required units of blood during a transfusion. Generally, the patients having high ISS, low GCS and increased need of blood units have poor outcome. In KSA, limited studies have been conducted on the topic of interest. This study was carried out to input new data to the literature review regarding the severity (ISS, GCS and units of blood transfusion) and management of TSIs and SCIs in Saudi individuals.

## Methods

This topic was chosen for in-depth research to fulfill the aim of determining the severity and neurosurgical management of traumatic spinal fractures in the Kingdom of Saudi Arabia (KSA). To the best of our knowledge, no such research has been conducted on this scale within KSA thus far. A comprehensive search was conducted until April 2018 on the PubMed, OVID Embase, OVID Medline and Cochrane databases with the following search terms: spinal fracture, traumatic spin and Saudi Arabia. One such study was found, conducted by El-Awad *et al*. in 2002, which looked at the treatment of thoracolumbar fractures at the Armed Forces Hospital, Riyadh, KSA between 1989 and 1999. No other studies which had a primary focus on the severity and neurosurgical management of traumatic spinal fractures could be identified from the literature review. As the literature is sparse in this domain, we set out on a prospective study conducted in the period from September 2016 to December 2017. For this prospective study, we recruited 120 patients, using a stringent set of inclusion and exclusion criteria. We arrived at an appropriate sample size of 120, after conducting a statistical power analysis. Utilizing the Clopper-Pearson formula, this study required us to recruit at least 12 patients to achieve a 95% confidence level together with a 5% margin of error. The inclusion criteria were as follows: male and female genders, all ages, provision of informed consent, living in Saudi Arabia, having presented to the King Khalid Hospital and Prince Sultan Center for Health Services, and having a primary or secondary diagnosis of traumatic spinal injury with neurological sequelae. The exclusion criteria were as follows: previously treated traumatic spinal injury, patients already admitted to an inpatient facility, and minors who had failed to provide legal guardian consent to take part in this study. All 120 patients were followed up from the point of triage to admission and discharge. We analysed the clinical notes of the 120 patients to determine the severity of their traumatic spinal injuries, the neurosurgical management carried out, as well as other prognosticating factors such as blood transfusion and the Glasgow Coma Scale (GCS). Furthermore, we also classified neurosurgical management into conservative management and surgical management. Within surgical management, we categorized patients into the following management pathways-decompression of the spinal cord, repair of the spinal cord, craniotomy and evacuation, and cervical collar with fixation. The data collected was analysed anonymously, and the confidentiality of all participants was respected. Collated data was entered manually by 3 independent authors into Microsoft Excel, and SPSS 22.0 was used for the statistical analysis. The threshold for statistical significance was deemed to be 0.05 or less, using independent sample t test.

## Results

**Figure 1:**
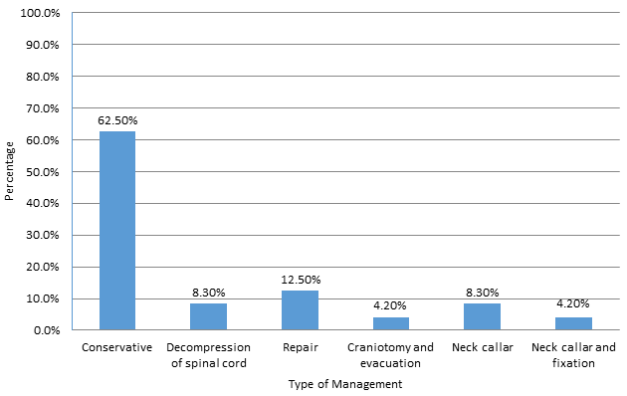
Management of traumatic spinal injuries

A total of 120 patients presented to the King Khalid Hospital and Prince Sultan Center for Health Services between September 2016 and December 2017 with traumatic spinal injuries and neurologic sequelae. Most of the patients were young adults and adolescents under the age of 40 (n = 96). There was a male preponderance of 84.1%; this was an expected finding given that most vehicle drivers in KSA are male. 26.6% and 54.2% patients required ICU admission and referred to other hospitals, respectively. These figures can be referenced in Table 1, Table 2, Table 3, Table 4, Table 5, Table 6 below. With respect to spinal injury stratification, out of the 120 patients who were recruited for this study, 55 patients had cervical spine fractures, 10 patients had cervical lacerations, 85 patients had thoracolumbar spinal fractures, and 10 patients had thoracolumbar spinal lacerations. 35 patients had other fractures documented. These figures may be referenced from Tables Table 1, Table 2, Table 3, Table 4, Table 5, Table 6 below. Injury severity scores (ISS) were documented for all injuries sustained by the 120 patients. The ISS is an established medical score utilized in the assessment of trauma severity, and it correlates with mortality, morbidity and length of inpatient hospitalization time post-trauma. In descending order, patients who sustained thoracolumbar spinal lacerations had the highest mean ISS of 46.5, followed by other fractures with a mean ISS of 38.86, followed by cervical lacerations with a mean ISS of 33.50, followed by thoracolumbar spinal fractures with a mean ISS of 33.24, and lastly cervical fractures with a mean ISS of 32.73. The mean ISS was found to be significantly higher in patients with a thoracolumbar spinal fracture (p<0.05). These figures can be referenced in tables Table 2, Table 3, Table 4, Table 5, Table 6 below. GCS was also documented for all 120 patients. The GCS is a 15-point neurological scale which objectively records the conscious state of a patient during the initial assessment. In ascending order, patients who sustained cervical spine lacerations had the lowest mean GCS of 9.50, followed by other fractures with a mean GCS of 10.71, followed by thoracolumbar spinal lacerations with a mean GCS of 12.00, followed by cervical spine fractures with a mean GCS of 12.18 and lastly thoracolumbar spinal fractures with a mean GCS of 12.71. The GCS was significantly lower in patients who sustained a cervical spine laceration or a cervical spine fracture (p<0.05). These figures can be referenced in Table 2, Table 3, Table 4, Table 5, Table 6 below. All 120 patients were followed up to assess the management of their traumatic spinal injuries. 66.6% (n= 80) of all patients were managed conservatively, whereas the remaining 33.3% (n=40) were managed surgically (Figure 1). Of the surgically managed patients, 25% underwent surgical decompression of the spinal cord, 37.5% underwent surgical repair of the spinal cord, 12.5% underwent a craniotomy and evacuation, and 12.5% underwent cervical collar placement and fixation. These figures can be referenced in Table 1 below. 26.6% of patients required an admission to the ICU, and 54.2% of patients were referred to another hospital for further management.

**Table 1:** Demographic breakdown of data

	Frequency	Percent
0-20	18	15%
21-30	45	37.5%
31-40	33	27.5%
41-50	15	12.5%
>50	9	7.5%
Female	19	15.8%
Male	101	84.1%
Yes	32	26.6%
No	88	73.3%
Yes	65	54.2%
No	55	45.8%

**Table 2:** ISS, GCS and blood transfusion in the patients with neck fractures and neck lacerations

ISS GCS blood transfusion * neck fracture					ISS GCS blood transfusion * neck laceration				
Type of injury		Mean	N	Std. Deviation	Type of injury		Mean	N	Std. Deviation
Neck Fracture (ISS)	No	28.85	65	11.838	Neck laceration (ISS)	No	30.36	110	14.880
	Yes	32.73	55	16.595		Yes	33.50	10	2.635
P value	0.692				P value	0.122			
Neck fracture (GCS)	No	13.65	65	2.888	Neck laceration (GCS)	No	13.32	110	3.020
	Yes	12.18	55	3.672		Yes	9.50	10	4.743
P value	0.000*				P value	0.000*			
Blood transfusion	No		65		Blood transfusion	No		110	
	Yes		55			Yes		10	
P value	1.000				P value	0.000*			

* Indicates statistical significance

**Table 3:** ISS, GCS and blood transfusion in the patients with spine injuries

Injury Type	Mean	N	Std. Deviation
No	24.29	35	9.529
Yes	33.24	85	15.125
**P value**	0.000*	-	-
No	14.29	35	1.045
Yes	12.47	85	3.797
**P value**	**0.226**	-	-
**BLOOD TRANSFUSION**	-	-	-
No	-	35	-
Yes	-	85	-
**P value**	0.037*	-	-

* Indicates statistical significance

**Table 4:** ISS, GCS and blood transfusion in the patients with spine fractures

Injury Type	Mean	N	Std. Deviation
No	29*.86	35	14.506
Yes	30.94	85	14.270
**P value**	**0.278**	-	-
No	13.71	35	1.506
Yes	12.71	85	3.823
**P value**	**0.420**	-	-
**BLOOD TRANSFUSION**	-	-	-
No	-	35	-
Yes	-	85	-
**P value**	**0.849**	-	-

**Table 5:** ISS, GCS and blood transfusion in the patients with spinal lacerations

Injury Type	Mean	N	Std. Deviation
No	29.18	110	13.693
Yes	46.50	10	11.068
**P value**	0.001*	-	-
No	13.09	110	3.466
Yes	12.00	10	1.054
**P value**	0.001*	-	-
**BLOOD TRANSFUSION**	**-**	-	-
No	**-**	110	-
Yes	**-**	10	-
**P value**	**0.211**	-	-

* Indicates statistical significance

**Table 6:** ISS, GCS and blood transfusion in the patients with other fractures

Injury Type	Mean	N	Std. Deviation
No	27.24	85	12.815
Yes	38.86	35	14.496
**P value**	0.000*	-	-
No	13.94	85	2.301
Yes	10.71	35	4.295
**P value**	0.000*	-	-
**BLOOD TRANSFUSION**	**-**	-	-
No	-	85	-
Yes	-	35	-
**P value**	**0.342**	-	-

* Indicates statistical significance

## Discussion

Traumatic spinal cord injury (TSCI) is devastating, and the neurological sequelae associated with it constitute a significant burden to the affected patient, and the healthcare system as a whole [11]. The true incidence of TSCI in KSA is challenging to derive, as the literature concerning it is sparse. Asirvatham and Zamzami [12] conducted a literature review on TSCI in KSA in 2013 and identified a study which was conducted on 307 patients between 2003 and 2008, the incidence of TSCI was reported as 2.1 per million [13]. With respect to aetiology, road traffic accidents represent the major cause of TSCI in KSA, especially in young adults. This is concordant with the findings from our study; 100% of the 120 patients studied were involved in an RTA, and a staggering 80% of the 120 patients studied were young adults and adolescents under the age of 40. Furthermore, there was a male preponderance of 84.1%; this is unsurprising, as most vehicle drivers in KSA are male. This is because KSA is uniquely positioned in that it is the only nation globally that legally precludes women from driving. In our study, we found that 54.1% of our patients sustained cervical injuries, whereas 79.2% of them sustained thoracolumbar injuries. This is congruent with findings from a recent meta-analysis which looked at the epidemiological characteristics of TSCI in the MENA region [14]. After analysing 29 studies, the authors found that injury at the cervical level had a random pooled estimate of 31%, whereas that of thoracolumbar injury was 71%.

However, our findings are not in keeping with another study conducted in 2004, which revealed 43.9% with cervical injuries and, equally, 43.9% with thoracolumbar injury [15]. This difference can be explained in two primary ways. First, the anatomical level of spinal cord injury is difficult to localise without the clinical examination or imaging of patients, and the ascertained level of spinal injury may very well depend on the skill of the examiner, or the skill of the radiologist interpreting the imaged spine. Secondly, the mechanisms of RTA may determine the type (high vs. low) of SCI. Our study is limited in that the mechanisms leading to the 120 RTAs were not collated and analysed. Our results demonstrated that GCS was significantly lower in patients who had sustained cervical spine injuries. This result is expected, as cervical injuries are 'high' SCIs, and therefore have a higher chance of concomitant traumatic brain injury (TBI). This is a finding which was substantiated in a prospective study conducted in 2008. The authors examined the incidence and severity of concomitant TBI in patients with TSCI; cervical SCI was associated with greater rates of TBI [16]. This study's findings confirm that: injuries to cervical and thoracolumbar regions are most commonly involved in RTAs; and following surgical repair and spinal decompression, most of these injuries only require conservative management, such as a neck collar. It follows that emergency medical staff should evaluate patients with TSIs carefully to facilitate immediate and customized treatment plans based on the severity of the injury (including ISS, GCS, and blood transfusions). To the best of our knowledge, we are the first study to demonstrate the management pathways undertaken by patients presenting with TSCI in KSA. A significant limitation of this study is that the factors which predict which management pathway (conservative vs. surgical) were not identified. Future studies could look at factors such as age, mechanism of RTA and presence of concomitant multisystemic injury as predictors of conservative vs. surgical management of TSCI.

## Conclusion

Trauma is an important and most common cause of spinal injuries (TSIs). Most of these injuries are managed conservatively; however, untreated TSIs or SCIs may lead to poor clinical outcome, especially if the cervical region is involved.

### What is known about this topic

- Road traffic accident is the most frequent cause of traumatic spinal injuries, contributing to 33.6% of all spinal fractures;
- Approximately, 768,473 cases of traumatic spinal injuries occur annually around the world;
- Kingdom of Saudi Arabia is one of the countries, which has high rates of spinal cord injuries.

### What this study adds

- Road traffic accidents are predominant among young male adults in Kingdom of Saudi Arabia;
- Most of these injuries are managed conservatively in Kingdom of Saudi Arabia;
- Untreated cervical traumatic spinal injuries or spinal cord injuries may lead to poor clinical outcome.

## Competing interests

The authors declare no competing interests.
